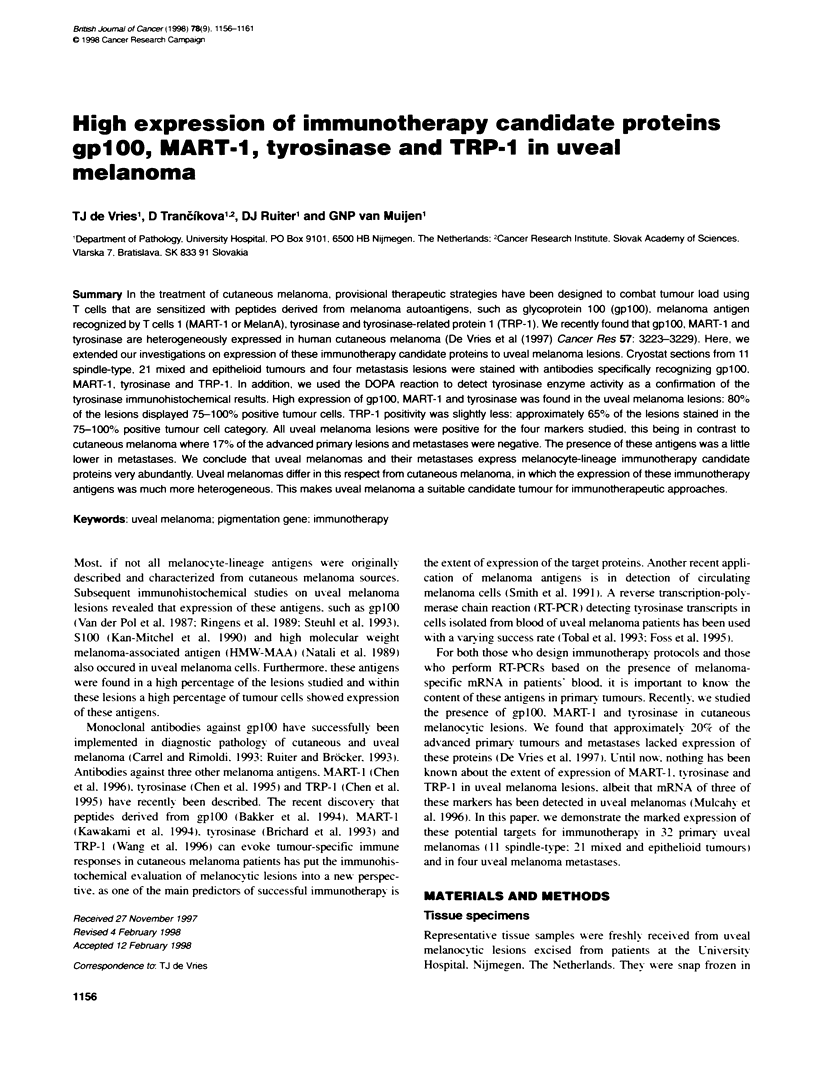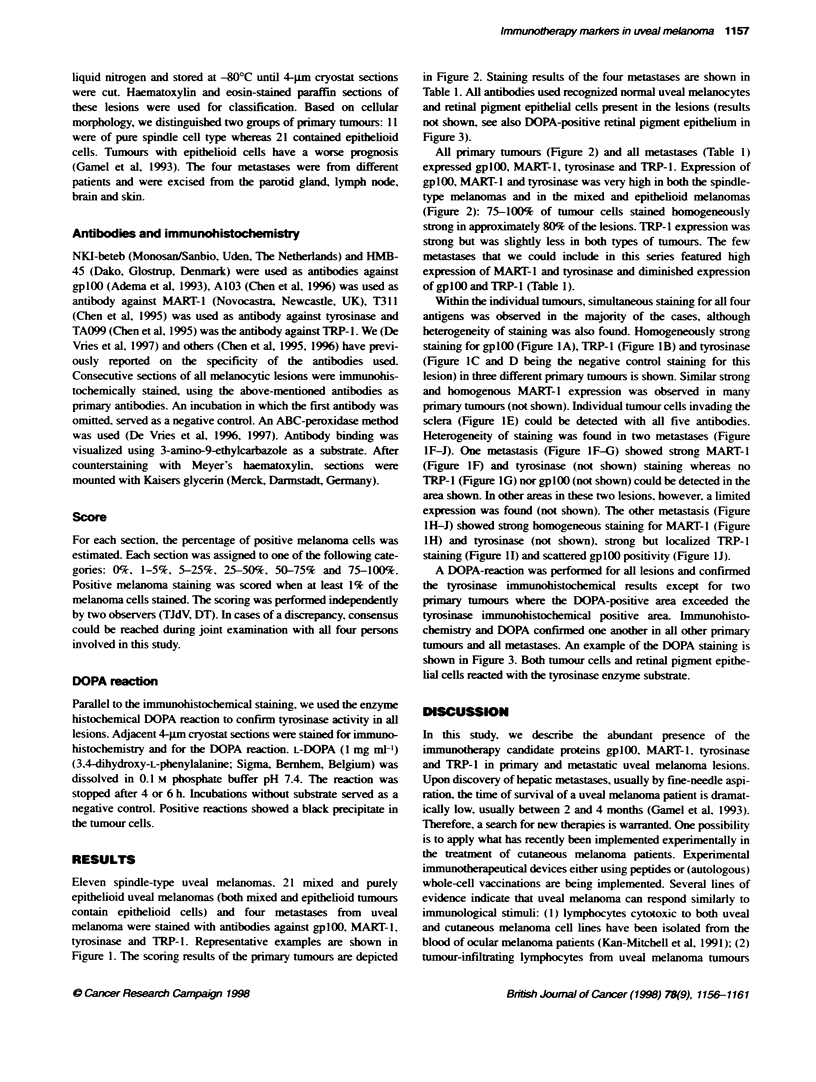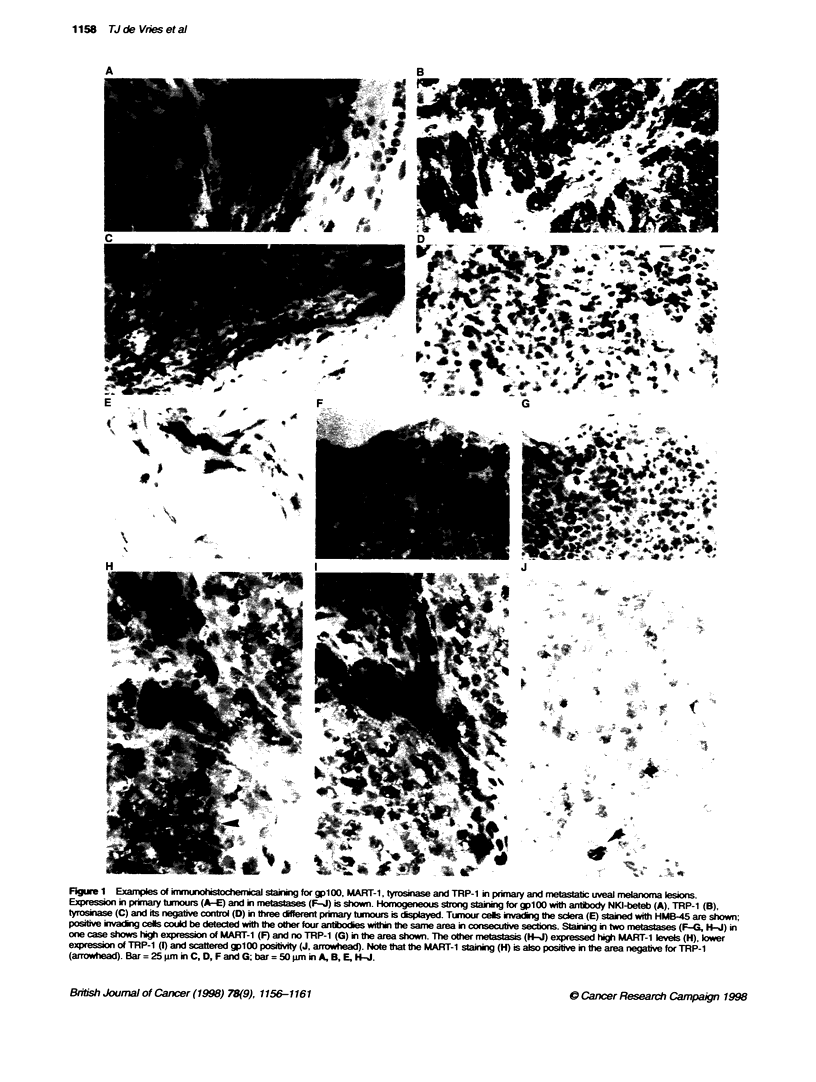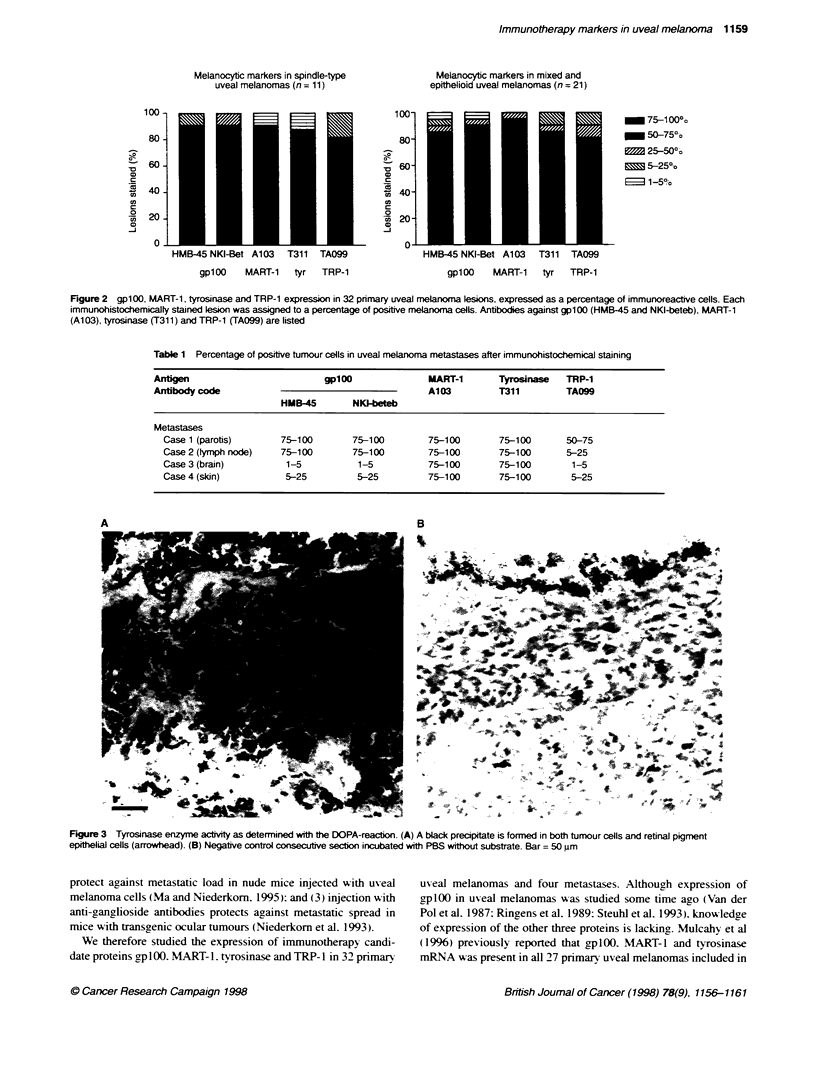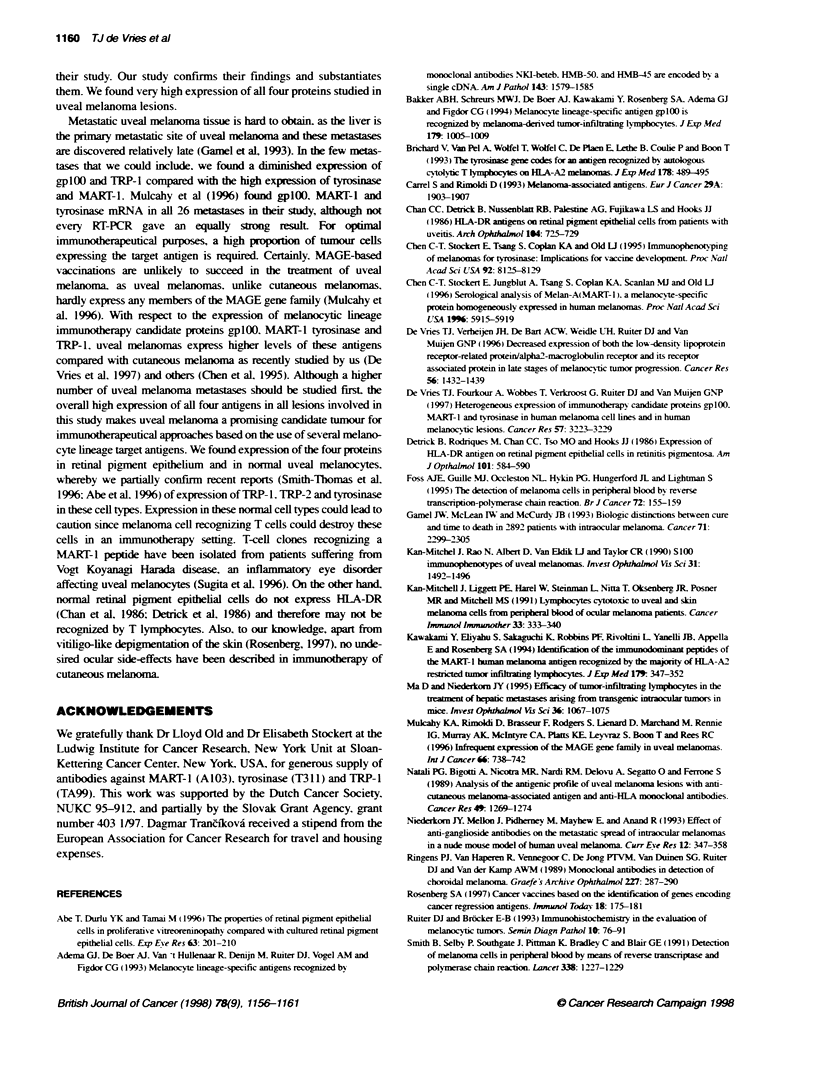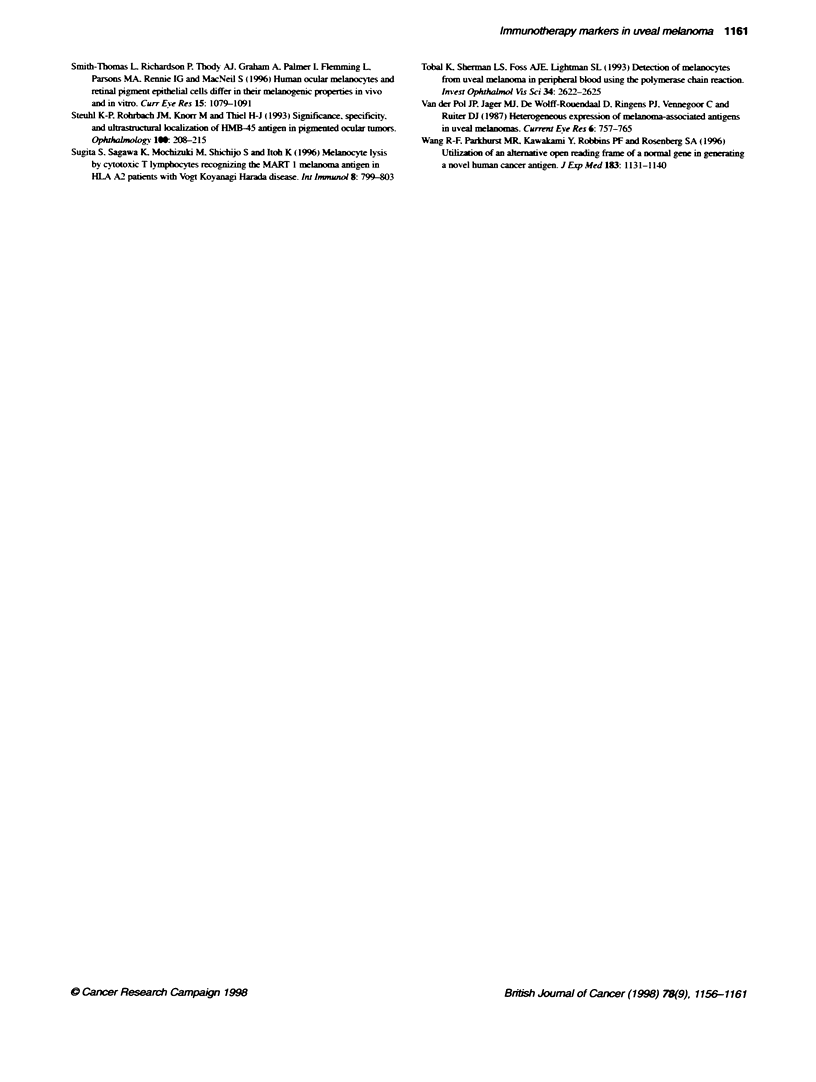# High expression of immunotherapy candidate proteins gp100, MART-1, tyrosinase and TRP-1 in uveal melanoma.

**DOI:** 10.1038/bjc.1998.646

**Published:** 1998-11

**Authors:** T. J. de Vries, D. Trancikova, D. J. Ruiter, G. N. van Muijen

**Affiliations:** Department of Pathology, University Hospital, Nijmegen, The Netherlands.

## Abstract

**Images:**


British Journal of Cancer (1998) 78(9). 1156–1161
© 1998 Cancer Research Campaign

### High expression of immunotherapy candidate proteins gp100, MART-1, tyrosinase and TRP-1 in uveal melanoma

**TJ de Vries[1], D Trančíkova[1,2], DJ Ruiter[1] and GNP van Muijen[1]**

[1]Department of Pathology. University Hospital. PO Box 9101. 6500 HB Nijmegen. The Netherlands: [2]Cancer Research Institute. Slovak Academy of Sciences. Vlarska 7. Bratislava. SK 833 91 Slovakia

**Summary** In the treatment of cutaneous melanoma, provisional therapeutic strategies have been designed to combat tumour load using T cells that are sensitized with peptides derived from melanoma autoantigens, such as glycoprotein 100 (gp100), melanoma antigen recognized by T cells 1 (MART-1 or MelanA), tyrosinase and tyrosinase-related protein 1 (TRP-1). We recently found that gp100, MART-1 and tyrosinase are heterogeneously expressed in human cutaneous melanoma (De Vries et al (1997) *Cancer Res* **57**: 3223–3229). Here, we extended our investigations on expression of these immunotherapy candidate proteins to uveal melanoma lesions. Cryostat sections from 11 spindle-type, 21 mixed and epithelioid tumours and four metastasis lesions were stained with antibodies specifically recognizing gp100, MART-1, tyrosinase and TRP-1. In addition, we used the DOPA reaction to detect tyrosinase enzyme activity as a confirmation of the tyrosinase immunohistochemical results. High expression of gp100, MART-1 and tyrosinase was found in the uveal melanoma lesions: 80% of the lesions displayed 75–100% positive tumour cells. TRP-1 positivity was slightly less: approximately 65% of the lesions stained in the 75–100% positive tumour cell category. All uveal melanoma lesions were positive for the four markers studied, this being in contrast to cutaneous melanoma where 17% of the advanced primary lesions and metastases were negative. The presence of these antigens was a little lower in metastases. We conclude that uveal melanomas and their metastases express melanocyte-lineage immunotherapy candidate proteins very abundantly. Uveal melanomas differ in this respect from cutaneous melanoma, in which the expression of these immunotherapy antigens was much more heterogeneous. This makes uveal melanoma a suitable candidate tumour for immunotherapeutic approaches.

**Keywords**: uveal melanoma; pigmentation gene; immunotherapy

Most, if not all melanocyte-lineage antigens were originally described and characterized from cutaneous melanoma sources. Subsequent immunohistochemical studies on uveal melanoma lesions revealed that expression of these antigens, such as gp100 (Van der Pol et al. 1987; Ringens et al. 1989; Steuhl et al. 1993). S100 (Kan-Mitchel et al. 1990) and high molecular weight melanoma-associated antigen (HMW-MAA) (Natali et al. 1989) also occured in uveal melanoma cells. Furthermore, these antigens were found in a high percentage of the lesions studied and within these lesions a high percentage of tumour cells showed expression of these antigens.

Monoclonal antibodies against gp100 have successfully been implemented in diagnostic pathology of cutaneous and uveal melanoma (Carrel and Rimoldi, 1993; Ruiter and Bröcker, 1993). Antibodies against three other melanoma antigens, MART-1 (Chen et al. 1996), tyrosinase (Chen et al. 1995) and TRP-1 (Chen et al. 1995) have recently been described. The recent discovery that peptides derived from gp100 (Bakker et al. 1994), MART-1 (Kawakami et al. 1994), tyrosinase (Brichard et al. 1993) and TRP-1 (Wang et al. 1996) can evoke tumour-specific immune responses in cutaneous melanoma patients has put the immunohistochemical evaluation of melanocytic lesions into a new perspective, as one of the main predictors of successful immunotherapy is the extent of expression of the target proteins. Another recent application of melanoma antigens is in detection of circulating melanoma cells (Smith et al. 1991). A reverse transcription-polymerase chain reaction (RT-PCR) detecting tyrosinase transcripts in cells isolated from blood of uveal melanoma patients has been used with a varying success rate (Tobal et al. 1993; Foss et al. 1995).

For both those who design immunotherapy protocols and those who perform RT-PCRs based on the presence of melanoma-specific mRNA in patients' blood, it is important to know the content of these antigens in primary tumours. Recently, we studied the presence of gp100, MART-1 and tyrosinase in cutaneous melanocytic lesions. We found that approximately 20% of the advanced primary tumours and metastases lacked expression of these proteins (De Vries et al. 1997). Until now, nothing has been known about the extent of expression of MART-1, tyrosinase and TRP-1 in uveal melanoma lesions, albeit that mRNA of three of these markers has been detected in uveal melanomas (Mulcahy et al. 1996). In this paper, we demonstrate the marked expression of these potential targets for immunotherapy in 32 primary uveal melanomas (11 spindle-type; 21 mixed and epithelioid tumours) and in four uveal melanoma metastases.

### MATERIALS AND METHODS

### Tissue specimens

Representative tissue samples were freshly received from uveal melanocytic lesions excised from patients at the University Hospital, Nijmegen, The Netherlands. They were snap frozen in

*Received 27 November 1997*
*Revised 4 February 1998*
*Accepted 12 February 1998*

*Correspondence to:* TJ de Vries liquid nitrogen and stored at –80°C until 4-μm cryostat sections were cut. Haematoxylin and eosin-stained paraffin sections of these lesions were used for classification. Based on cellular morphology, we distinguished two groups of primary tumours: 11 were of pure spindle cell type whereas 21 contained epithelioid cells. Tumours with epithelioid cells have a worse prognosis (Gamel et al, 1993). The four metastases were from different patients and were excised from the parotid gland, lymph node, brain and skin.

### Antibodies and immunohistochemistry

NKI-beteb (Monosan/Sanbio, Uden, The Netherlands) and HMB-45 (Dako, Glostrup, Denmark) were used as antibodies against gp100 (Adema et al, 1993), A103 (Chen et al, 1996) was used as antibody against MART-1 (Novocastra, Newcastle, UK), T311 (Chen et al, 1995) was used as antibody against tyrosinase and TA099 (Chen et al, 1995) was the antibody against TRP-1. We (De Vries et al, 1997) and others (Chen et al, 1995, 1996) have previously reported on the specificity of the antibodies used. Consecutive sections of all melanocytic lesions were immunohistochemically stained, using the above-mentioned antibodies as primary antibodies. An incubation in which the first antibody was omitted, served as a negative control. An ABC-peroxidase method was used (De Vries et al, 1996, 1997). Antibody binding was visualized using 3-amino-9-ethylcarbazole as a substrate. After counterstaining with Meyer's haematoxylin, sections were mounted with Kaisers glycerin (Merck, Darmstadt, Germany).

### Score

For each section, the percentage of positive melanoma cells was estimated. Each section was assigned to one of the following categories: 0%, 1–5%, 5–25%, 25–50%, 50–75% and 75–100%. Positive melanoma staining was scored when at least 1% of the melanoma cells stained. The scoring was performed independently by two observers (TJdV, DT). In cases of a discrepancy, consensus could be reached during joint examination with all four persons involved in this study.

### DOPA reaction

Parallel to the immunohistochemical staining, we used the enzyme histochemical DOPA reaction to confirm tyrosinase activity in all lesions. Adjacent 4-μm cryostat sections were stained for immunohistochemistry and for the DOPA reaction. L-DOPA (1 mg $ml^{-1}$) (3,4-dihydroxy-L-phenylalanine; Sigma, Bernhem, Belgium) was dissolved in 0.1 M phosphate buffer pH 7.4. The reaction was stopped after 4 or 6 h. Incubations without substrate served as a negative control. Positive reactions showed a black precipitate in the tumour cells.

### RESULTS

Eleven spindle-type uveal melanomas, 21 mixed and purely epithelioid uveal melanomas (both mixed and epithelioid tumours contain epithelioid cells) and four metastases from uveal melanoma were stained with antibodies against gp100, MART-1, tyrosinase and TRP-1. Representative examples are shown in Figure 1. The scoring results of the primary tumours are depicted in Figure 2. Staining results of the four metastases are shown in Table 1. All antibodies used recognized normal uveal melanocytes and retinal pigment epithelial cells present in the lesions (results not shown, see also DOPA-positive retinal pigment epithelium in Figure 3).

All primary tumours (Figure 2) and all metastases (Table 1) expressed gp100, MART-1, tyrosinase and TRP-1. Expression of gp100, MART-1 and tyrosinase was very high in both the spindle-type melanomas and in the mixed and epithelioid melanomas (Figure 2): 75–100% of tumour cells stained homogeneously strong in approximately 80% of the lesions. TRP-1 expression was strong but was slightly less in both types of tumours. The few metastases that we could include in this series featured high expression of MART-1 and tyrosinase and diminished expression of gp100 and TRP-1 (Table 1).

Within the individual tumours, simultaneous staining for all four antigens was observed in the majority of the cases, although heterogeneity of staining was also found. Homogeneously strong staining for gp100 (Figure 1A), TRP-1 (Figure 1B) and tyrosinase (Figure 1C and D being the negative control staining for this lesion) in three different primary tumours is shown. Similar strong and homogenous MART-1 expression was observed in many primary tumours (not shown). Individual tumour cells invading the sclera (Figure 1E) could be detected with all five antibodies. Heterogeneity of staining was found in two metastases (Figure 1F–J). One metastasis (Figure 1F–G) showed strong MART-1 (Figure 1F) and tyrosinase (not shown) staining whereas no TRP-1 (Figure 1G) nor gp100 (not shown) could be detected in the area shown. In other areas in these two lesions, however, a limited expression was found (not shown). The other metastasis (Figure 1H–J) showed strong homogeneous staining for MART-1 (Figure 1H) and tyrosinase (not shown), strong but localized TRP-1 staining (Figure 1I) and scattered gp100 positivity (Figure 1J).

A DOPA-reaction was performed for all lesions and confirmed the tyrosinase immunohistochemical results except for two primary tumours where the DOPA-positive area exceeded the tyrosinase immunohistochemical positive area. Immunohistochemistry and DOPA confirmed one another in all other primary tumours and all metastases. An example of the DOPA staining is shown in Figure 3. Both tumour cells and retinal pigment epithelial cells reacted with the tyrosinase enzyme substrate.

### DISCUSSION

In this study, we describe the abundant presence of the immunotherapy candidate proteins gp100, MART-1, tyrosinase and TRP-1 in primary and metastatic uveal melanoma lesions. Upon discovery of hepatic metastases, usually by fine-needle aspiration, the time of survival of a uveal melanoma patient is dramatically low, usually between 2 and 4 months (Gamel et al. 1993). Therefore, a search for new therapies is warranted. One possibility is to apply what has recently been implemented experimentally in the treatment of cutaneous melanoma patients. Experimental immunotherapeutical devices either using peptides or (autologous) whole-cell vaccinations are being implemented. Several lines of evidence indicate that uveal melanoma can respond similarly to immunological stimuli: (1) lymphocytes cytotoxic to both uveal and cutaneous melanoma cell lines have been isolated from the blood of ocular melanoma patients (Kan-Mitchell et al, 1991); (2) tumour-infiltrating lymphocytes from uveal melanoma tumours

© Cancer Research Campaign 1998

**Figure 1** Examples of immunohistochemical staining for gp100, MART-1, tyrosinase and TRP-1 in primary and metastatic uveal melanoma lesions. Expression in primary tumours (**A–E**) and in metastases (**F–J**) is shown. Homogeneous strong staining for gp100 with antibody NKI-beteb (**A**), TRP-1 (**B**), tyrosinase (**C**) and its negative control (**D**) in three different primary tumours is displayed. Tumour cells invading the sclera (**E**) stained with HMB-45 are shown; positive invading cells could be detected with the other four antibodies within the same area in consecutive sections. Staining in two metastases (**F–G**, **H–J**) in one case shows high expression of MART-1 (**F**) and no TRP-1 (**G**) in the area shown. The other metastasis (**H–J**) expressed high MART-1 levels (**H**), lower expression of TRP-1 (**I**) and scattered gp100 positivity (**J**, arrowhead). Note that the MART-1 staining (**H**) is also positive in the area negative for TRP-1 (arrowhead). Bar = 25 μm in **C**, **D**, **F** and **G**; bar = 50 μm in **A**, **B**, **E**, **H–J**.

© Cancer Research Campaign 1998

**Figure 2** gp100, MART-1, tyrosinase and TRP-1 expression in 32 primary uveal melanoma lesions, expressed as a percentage of immunoreactive cells. Each immunohistochemically stained lesion was assigned to a percentage of positive melanoma cells. Antibodies against gp100 (HMB-45 and NKI-beteb), MART-1 (A103), tyrosinase (T311) and TRP-1 (TA099) are listed

**Table 1** Percentage of positive tumour cells in uveal melanoma metastases after immunohistochemical staining

| **Antigen** | **gp100** | | **MART-1** | **Tyrosinase** | **TRP-1** |
|---|---|---|---|---|---|
| **Antibody code** | **HMB-45** | **NKI-beteb** | **A103** | **T311** | **TA099** |
| Metastases | | | | | |
| Case 1 (parotis) | 75–100 | 75–100 | 75–100 | 75–100 | 50–75 |
| Case 2 (lymph node) | 75–100 | 75–100 | 75–100 | 75–100 | 5–25 |
| Case 3 (brain) | 1–5 | 1–5 | 75–100 | 75–100 | 1–5 |
| Case 4 (skin) | 5–25 | 5–25 | 75–100 | 75–100 | 5–25 |

**Figure 3** Tyrosinase enzyme activity as determined with the DOPA-reaction. (**A**) A black precipitate is formed in both tumour cells and retinal pigment epithelial cells (arrowhead). (**B**) Negative control consecutive section incubated with PBS without substrate. Bar = 50 μm protect against metastatic load in nude mice injected with uveal melanoma cells (Ma and Niederkorn, 1995); and (3) injection with anti-ganglioside antibodies protects against metastatic spread in mice with transgenic ocular tumours (Niederkorn et al, 1993).

We therefore studied the expression of immunotherapy candidate proteins gp100, MART-1, tyrosinase and TRP-1 in 32 primary uveal melanomas and four metastases. Although expression of gp100 in uveal melanomas was studied some time ago (Van der Pol et al, 1987; Ringens et al, 1989; Steuhl et al, 1993), knowledge of expression of the other three proteins is lacking. Mulcahy et al (1996) previously reported that gp100, MART-1 and tyrosinase mRNA was present in all 27 primary uveal melanomas included in

© *Cancer Research Campaign 1998*

their study. Our study confirms their findings and substantiates them. We found very high expression of all four proteins studied in uveal melanoma lesions.

Metastatic uveal melanoma tissue is hard to obtain, as the liver is the primary metastatic site of uveal melanoma and these metastases are discovered relatively late (Gamel et al, 1993). In the few metastases that we could include, we found a diminished expression of gp100 and TRP-1 compared with the high expression of tyrosinase and MART-1. Mulcahy et al (1996) found gp100, MART-1 and tyrosinase mRNA in all 26 metastases in their study, although not every RT-PCR gave an equally strong result. For optimal immunotherapeutical purposes, a high proportion of tumour cells expressing the target antigen is required. Certainly, MAGE-based vaccinations are unlikely to succeed in the treatment of uveal melanoma, as uveal melanomas, unlike cutaneous melanomas, hardly express any members of the MAGE gene family (Mulcahy et al, 1996). With respect to the expression of melanocytic lineage immunotherapy candidate proteins gp100, MART-1 tyrosinase and TRP-1, uveal melanomas express higher levels of these antigens compared with cutaneous melanoma as recently studied by us (De Vries et al, 1997) and others (Chen et al, 1995). Although a higher number of uveal melanoma metastases should be studied first, the overall high expression of all four antigens in all lesions involved in this study makes uveal melanoma a promising candidate tumour for immunotherapeutical approaches based on the use of several melanocyte lineage target antigens. We found expression of the four proteins in retinal pigment epithelium and in normal uveal melanocytes, whereby we partially confirm recent reports (Smith-Thomas et al, 1996; Abe et al, 1996) of expression of TRP-1, TRP-2 and tyrosinase in these cell types. Expression in these normal cell types could lead to caution since melanoma cell recognizing T cells could destroy these cells in an immunotherapy setting. T-cell clones recognizing a MART-1 peptide have been isolated from patients suffering from Vogt Koyanagi Harada disease, an inflammatory eye disorder affecting uveal melanocytes (Sugita et al, 1996). On the other hand, normal retinal pigment epithelial cells do not express HLA-DR (Chan et al, 1986; Detrick et al, 1986) and therefore may not be recognized by T lymphocytes. Also, to our knowledge, apart from vitiligo-like depigmentation of the skin (Rosenberg, 1997), no undesired ocular side-effects have been described in immunotherapy of cutaneous melanoma.

### ACKNOWLEDGEMENTS

We gratefully thank Dr Lloyd Old and Dr Elisabeth Stockert at the Ludwig Institute for Cancer Research, New York Unit at Sloan-Kettering Cancer Center, New York, USA, for generous supply of antibodies against MART-1 (A103), tyrosinase (T311) and TRP-1 (TA99). This work was supported by the Dutch Cancer Society, NUKC 95–912, and partially by the Slovak Grant Agency, grant number 403 1/97. Dagmar Trančíková received a stipend from the European Association for Cancer Research for travel and housing expenses.

### REFERENCES

Abe T, Durlu YK and Tamai M (1996) The properties of retinal pigment epithelial cells in proliferative vitreoreninopathy compared with cultured retinal pigment epithelial cells. *Exp Eye Res* **63**: 201–210

Adema GJ, De Boer AJ, Van 't Hullenaar R, Denijn M, Ruiter DJ, Vogel AM and Figdor CG (1993) Melanocyte lineage-specific antigens recognized by monoclonal antibodies NKI-beteb, HMB-50, and HMB-45 are encoded by a single cDNA. *Am J Pathol* **143**: 1579–1585

Bakker ABH, Schreurs MWJ, De Boer AJ, Kawakami Y, Rosenberg SA, Adema GJ and Figdor CG (1994) Melanocyte lineage-specific antigen gp100 is recognized by melanoma-derived tumor-infiltrating lymphocytes. *J Exp Med* **179**: 1005–1009

Brichard V, Van Pel A, Wolfel T, Wolfel C, De Plaen E, Lethe B, Coulie P and Boon T (1993) The tyrosinase gene codes for an antigen recognized by autologous cytolytic T lymphocytes on HLA-A2 melanomas. *J Exp Med* **178**: 489–495

Carrel S and Rimoldi D (1993) Melanoma-associated antigens. *Eur J Cancer* **29A**: 1903–1907

Chan CC, Detrick B, Nussenblatt RB, Palestine AG, Fujikawa LS and Hooks JJ (1986) HLA-DR antigens on retinal pigment epithelial cells from patients with uveitis. *Arch Ophthalmol* **104**: 725–729

Chen C-T, Stockert E, Tsang S, Coplan KA and Old LJ (1995) Immunophenotyping of melanomas for tyrosinase: Implications for vaccine development. *Proc Natl Acad Sci USA* **92**: 8125–8129

Chen C-T, Stockert E, Jungblut A, Tsang S, Coplan KA, Scanlan MJ and Old LJ (1996) Serological analysis of Melan-A(MART-1), a melanocyte-specific protein homogeneously expressed in human melanomas. *Proc Natl Acad Sci USA* **1996**: 5915–5919

De Vries TJ, Verheijen JH, De Bart ACW, Weidle UH, Ruiter DJ and Van Muijen GNP (1996) Decreased expression of both the low-density lipoprotein receptor-related protein/alpha2-macroglobulin receptor and its receptor associated protein in late stages of melanocytic tumor progression. *Cancer Res* **56**: 1432–1439

De Vries TJ, Fourkour A, Wobbes T, Verkroost G, Ruiter DJ and Van Muijen GNP (1997) Heterogeneous expression of immunotherapy candidate proteins gp100, MART-1 and tyrosinase in human melanoma cell lines and in human melanocytic lesions. *Cancer Res* **57**: 3223–3229

Detrick B, Rodriques M, Chan CC, Tso MO and Hooks JJ (1986) Expression of HLA-DR antigen on retinal pigment epithelial cells in retinitis pigmentosa. *Am J Opthalmol* **101**: 584–590

Foss AJE, Guille MJ, Occleston NL, Hykin PG, Hungerford JL and Lightman S (1995) The detection of melanoma cells in peripheral blood by reverse transcription-polymerase chain reaction. *Br J Cancer* **72**: 155–159

Gamel JW, McLean IW and McCurdy JB (1993) Biologic distinctions between cure and time to death in 2892 patients with intraocular melanoma. *Cancer* **71**: 2299–2305

Kan-Mitchel J, Rao N, Albert D, Van Eldik LJ and Taylor CR (1990) S100 immunophenotypes of uveal melanomas. *Invest Ophthalmol Vis Sci* **31**: 1492–1496

Kan-Mitchell J, Liggett PE, Harel W, Steinman L, Nitta T, Oksenberg JR, Posner MR and Mitchell MS (1991) Lymphocytes cytotoxic to uveal and skin melanoma cells from peripheral blood of ocular melanoma patients. *Cancer Immunol Immunother* **33**: 333–340

Kawakami Y, Eliyahu S, Sakaguchi K, Robbins PF, Rivoltini L, Yanelli JB, Appella E and Rosenberg SA (1994) Identification of the immunodominant peptides of the MART-1 human melanoma antigen recognized by the majority of HLA-A2 restricted tumor infiltrating lymphocytes. *J Exp Med* **179**: 347–352

Ma D and Niederkorn JY (1995) Efficacy of tumor-infiltrating lymphocytes in the treatment of hepatic metastases arising from transgenic intraocular tumors in mice. *Invest Ophthalmol Vis Sci* **36**: 1067–1075

Mulcahy KA, Rimoldi D, Brasseur F, Rodgers S, Lienard D, Marchand M, Rennie IG, Murray AK, McIntyre CA, Platts KE, Leyvraz S, Boon T and Rees RC (1996) Infrequent expression of the MAGE gene family in uveal melanomas. *Int J Cancer* **66**: 738–742

Natali PG, Bigotti A, Nicotra MR, Nardi RM, Delovu A, Segatto O and Ferrone S (1989) Analysis of the antigenic profile of uveal melanoma lesions with anti-cutaneous melanoma-associated antigen and anti-HLA monoclonal antibodies. *Cancer Res* **49**: 1269–1274

Niederkorn JY, Mellon J, Pidherney M, Mayhew E, and Anand R (1993) Effect of anti-ganglioside antibodies on the metastatic spread of intraocular melanomas in a nude mouse model of human uveal melanoma. *Curr Eye Res* **12**: 347–358

Ringens PJ, Van Haperen R, Vennegoor C, De Jong PTVM, Van Duinen SG, Ruiter DJ and Van der Kamp AWM (1989) Monoclonal antibodies in detection of choroidal melanoma. *Graefe's Archive Ophthalmol* **227**: 287–290

Rosenberg SA (1997) Cancer vaccines based on the identification of genes encoding cancer regression antigens. *Immunol Today* **18**: 175–181

Ruiter DJ and Bröcker E-B (1993) Immunohistochemistry in the evaluation of melanocytic tumors. *Semin Diagn Pathol* **10**: 76–91

Smith B, Selby P, Southgate J, Pittman K, Bradley C and Blair GE (1991) Detection of melanoma cells in peripheral blood by means of reverse transcriptase and polymerase chain reaction. *Lancet* **338**: 1227–1229

© Cancer Research Campaign 1998

Smith-Thomas L, Richardson P, Thody AJ, Graham A, Palmer I, Flemming L, Parsons MA, Rennie IG and MacNeil S (1996) Human ocular melanocytes and retinal pigment epithelial cells differ in their melanogenic properties in vivo and in vitro. *Curr Eye Res* **15**: 1079–1091

Steuhl K-P, Rohrbach JM, Knorr M and Thiel H-J (1993) Significance, specificity, and ultrastructural localization of HMB-45 antigen in pigmented ocular tumors. *Ophthalmology* **100**: 208–215

Sugita S, Sagawa K, Mochizuki M, Shichijo S and Itoh K (1996) Melanocyte lysis by cytotoxic T lymphocytes recognizing the MART 1 melanoma antigen in HLA A2 patients with Vogt Koyanagi Harada disease. *Int Immunol* **8**: 799–803

Tobal K, Sherman LS, Foss AJE, Lightman SL (1993) Detection of melanocytes from uveal melanoma in peripheral blood using the polymerase chain reaction. *Invest Ophthalmol Vis Sci* **34**: 2622–2625

Van der Pol JP, Jager MJ, De Wolff-Rouendaal D, Ringens PJ, Vennegoor C and Ruiter DJ (1987) Heterogeneous expression of melanoma-associated antigens in uveal melanomas. *Current Eye Res* **6**: 757–765

Wang R-F, Parkhurst MR, Kawakami Y, Robbins PF and Rosenberg SA (1996) Utilization of an alternative open reading frame of a normal gene in generating a novel human cancer antigen. *J Exp Med* **183**: 1131–1140

© Cancer Research Campaign 1998